# Autophagy involved in lipopolysaccharide-induced foam cell formation is mediated by adipose differentiation-related protein

**DOI:** 10.1186/1476-511X-13-10

**Published:** 2014-01-09

**Authors:** Xuyang Feng, Yuan Yuan, Chao Wang, Jun Feng, Zuyi Yuan, Xiumin Zhang, Wen Sui, Peizhen Hu, Pengfei Zheng, Jing Ye

**Affiliations:** 1 Department of cardiology, Xijing Hospital, The Fourth Military Medical University, Xi’an 710032, China; 2 Department of Pathology, Xijing Hospital, The Fourth Military Medical University, Xi’an 710032, China; 3 Department of Cerebral vessels, First Affiliated Hospital of Medical College, Xi’an Jiaotong University, Xi’an 710061, China; 4 Department of cardiology, First Affiliated Hospital of Medical College, Xi’an Jiaotong University, Xi’an 710061, China

**Keywords:** Lipopolysaccharide, Lipid droplet, Foam cell, Autophagy, ADRP

## Abstract

**Background:**

Autophagy is an essential process for breaking down macromolecules and aged/damaged cellular organelles to maintain cellular energy balance and cellular nutritional status. The idea that autophagy regulates lipid metabolism is an emerging concept with important implications for atherosclerosis. However, the potential role of autophagy and its relationship with lipid metabolism in foam cell formation remains unclear. In this study, we found that autophagy was involved in the lipopolysaccharide (LPS)-induced the formation of foam cells and was at least partially dependent on adipose differentiation-related protein (ADRP).

**Method:**

Foam cell formation was evaluated by Oil red O staining. Autophagic activity was determined by immunofluorescence and Western blotting. ADRP gene expression of ADRP was examined by real-time PCR (RT-PCR). The protein expression of ADRP and LC3 was measured using Western blotting analysis. Intracellular cholesterol and triglyceride levels in foam cells were quantitatively measured by enzymatic colorimetric assays.

**Results:**

LPS promoted foam cell formation by inducing lipid accumulation in macrophages. The activation of autophagy with rapamycin (Rap) decreased intracellular cholesterol and triglyceride levels, whereas the inhibition of autophagy with 3-methyladenine (3MA) enhanced the accumulation of lipid droplets. Overexpression of ADRP alone increased the formation of foam cells and consequently autophagic activity. In contrast, the inhibitory effects of ADRP activity with siRNA suppressed the activation of autophagy. Taken together, we propose a novel role for ADRP in the regulation of macrophage autophagy during LPS stimulation.

**Conclusion:**

We defined a new molecular pathway in which LPS-induced foam cell formation is regulated through autophagy. These findings facilitate the understanding of the role of autophagy in the development of atherosclerosis.

## Introduction

Atherosclerosis is a chronic lipid metabolism disorder characterized by the deposition of excess lipids in large arteries. Macrophages take up excessive modified lipoproteins, leading to the accumulation of intracellular cholesterol and triglycerides in the form of lipid droplets and the formation of foam cells, which appear to be a hallmark of the atherosclerosis [1,2]. Therefore, understanding the potential mechanism of foam cell formation is critical for elucidating the pathogenesis of atherosclerosis and treatment.

Autophagy is a highly regulated intracellular degradation process that mediates the clearance of cytoplasmic proteins, certain pathogens and organelles [3]. When autophagy is initiated, double-membraned autophagosomes form randomly in the cytoplasm and eventually fuse with a lysosome to form an autolysosome where the contents are degraded and recycled for protein synthesis [4]. During the process of autophagic membrane formation, microtubule-associated protein 1 light chain 3 (LC3) is conjugated to a lipid to generate LC3-II that is one of the best characterized structural components of the autophagosomes [5].

Recently, LPS, a potential mediator of inflammatory responses, has been found to induce the macrophage-derived foam cell formation in vitro and promote the development of atherosclerotic plaque in vivo [6,7]. In addition, LPS could increase the expression of ADRP, a lipid droplet-associated proteins, as part of a coordinated change in macrophage physiology [8]. Studies have indicated that LPS induces autophagy in macrophages [6,9], and autophagy has been shown to be activated in advanced (late stage) atherosclerotic plaques [10,11], suggesting that autophagy may play an important role in LPS-induced foam cell formation.

Despite the increasing interest in the phenomenon of autophagy, the role of autophagy in atherosclerotic development and the relationship between autophagy and lipid accumulation have not been established. In this study, we describe a role for autophagy in controlling foam cell formation, which appears to be dependent on ADRP.

## Materials and methods

### Cell lines, antibodies and chemicals

The human monocytic cell line THP-1 was purchased from the American Type Culture Collection (ATCC). Antibodies against LC3 (Cell signaling technology), ADRP (Abcam) and β-actin (Sigma) were used. LPS, phorbol 12-myristate 13-acetate (PMA), oxidized low density lipoprotein (oxLDL), rapamycin (Rap), and 3-methyladenine (3MA) were purchased from Sigma.

### Cell culture and foam cell formation evaluation by Oil red O staining

Human THP-1 cells were seeded into six-well plates at 2 × 10^5^ cells per well in RPMI1640 medium containing 10% fetal bovine serum (FBS), and were maintained at 37°C in a humidified incubator in a 95% air plus 5% CO_2_ atmosphere. The THP-1 monocytes were treated by the addition of 100 nM PMA for 24 h to facilitate monocytes differentiation into macrophages. After PMA treatment, the adherent macrophages were transformed into foam cells by incubation with 50 μg/ml oxLDL for 24 h. The cells were then treated with Rap (10 μM) or 3MA (10 μM) in the absence or presence of LPS (1 μg/ml) for 24 h. Cultured foam cells were then washed once with phosphate-buffered saline (PBS) and fixed in 4% paraformaldehyde for 30 min. After rinsing with 60% isopropanol, foam cells were stained with 0.3% Oil Red O in 60% isopropanol for 15 min and then washed again with 60% isopropanol again. Afterward, foam cells were counterstained with hematoxylin for 3 min. After washing with water, foam cells were photographed with a microscope at 400× magnification.

### Measurement of intracellular cholesterol and triglyceride levels

The content of cholesterol and triglycerides in macrophage cells was quantitatively measured by enzymatic colorimetric assays with the kits from Wako (Richmond, VA) according to the manufacturer’s protocols. Briefly, 20 μl of each sample (cell lysate), standard (cholesterol 200 mg/dL) and blank (distilled water) were added into the pre-labeled tubes and added with 2 ml of color reagent (containing cholesterol oxidase, peroxidase, cholesterol ester hydrolase, ascorbate oxidase, 4-aminoantipyrine and DAOS). These reactions were mixed well and incubated at 37°C for 5 min. Finally, the measurements of the absorbance of the samples and standard against the blank were performed at 600 nm. The total cholesterol and triglycerides corresponding to the absorbance of samples were calculated from the standard calibration curve. The concentration of cellular proteins from these cells was measured with a protein assay kit from Bio-Rad (Hercules, CA).

### Transfection and establishment of stable cell lines

THP-1 cells were transfected with 2 μg of the purified recombinant plasmid pEGFP-LC3 using the Lipofectamine 2000 transfection reagent (Invitrogen Life Technologies). THP-1 cells stably expressing GFP-LC3 were selected with G418 for 3-4 weeks. The pEGFP vector was used as the control for GFP-LC3.

### Autophagy assays

Autophagy was evaluated in cells by fluorescence microscopy, or immunoblotting. In fluorescence microscopy experiments, autophagy was evaluated by examining the punctate forms (type II) of the autophagy marker LC3. Experiments examined either GFP-LC3 or endogenous LC3 stained with the LC3 antibody. Quantitation of autophagy was performed based on the percentage of GFP-LC3-positive autophagic vacuoles or cells with LC3 punctate dots. In all experiments, a minimum of 100 cells per sample was counted, and duplicate or triplicate samples were counted per experimental condition [12]. Statistical analysis was performed using a two-tailed Student’s *t* test.

### Overexpression or knockdown of ADRP in THP-1 macrophages

After induced with PMA, the THP-1 cells were transiently transfected with 2 μg of pCMV5-ADRP or 100 nM siRNA against ADRP with Lipofectamine 2000 transfection reagents (Invitrogen Life Technologies) according to instructions of the manufacturer. Transfection mixtures were added to cells in Opti-MEM medium for 16 h before media was replaced with regular RPMI 1640 media supplemented with 10% FBS.

### Real-time PCR

At the end of incubation, total RNA was isolated with TRIzol (TAKARA) according to the manufacturer’s instructions. Two micrograms of total RNA were reverse transcribed into cDNA. The cDNA was subsequently subjected to SYBR Green based real-time PCR using ABI 7500 real time PCR System (Applied Biosystems, Alameda, CA, USA). Primers used in real-time PCR are shown as in Table 1. The gene expression was normalized against the expression of β-actin.

**Table 1:** Primers used for real time PCR

**Gene**	**Species**	**Sequence (5′-3′)**
ADRP	Human	Forward: 5′-TTGCAGTTGCCAATACCTATGC-3′
		Reverse: 5′-CCAGTCACAGTAGTCGTCACA-3′
β-actin	Human	Forward: 5′-CATGTACGTTGCTATCCAGGC-3′
		Reverse: 5′-CTCCTTAATGTCACGCACGAT-3′

### Western blot analysis

Cells were lysed in RIPA buffer (25 mmol/L Tris–HCl pH 7.6, 150 mmol/L NaCl, 1% NP-40, 1% deoxycholate, 1% Triton X-100, 0.5% SDS, 2 mmol/L EDTA, 0.5 mmol/L PMSF, protease inhibitor cocktail). Protein was quantified with Bio-Rad protein assay reagents. Prestained molecular markers (Fermentas) were used to estimate the molecular weight of samples. Proteins were transferred to PVDF membranes (Millipore), and after incubation with primary and secondary antibodies, were detected by ECL regents (Amersham Biosciences). Bands were normalized to β-actin and expressed as a percent of control.

### Statistical analysis

Values were shown as the mean ± SEM of measurements of at least three independently performed experiments to avoid possible variation of the cell cultures. For statistical analyses, Student’s *t* test was employed and *P* < 0.05 was considered to be statistically significant.

## Results

### LPS promotes foam cell formation by inducing lipid accumulation in macrophages

**Figure 1:**
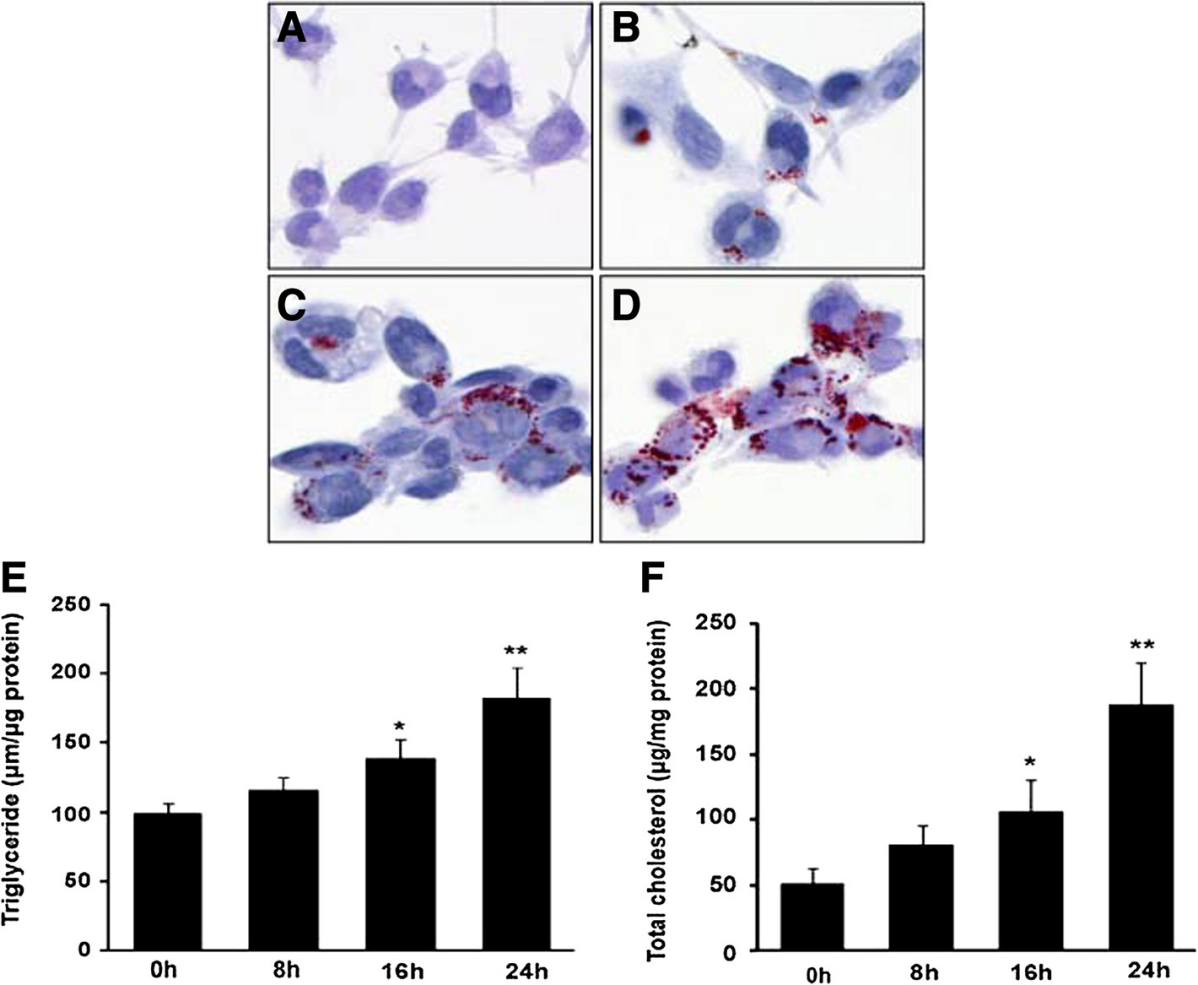
**LPS enhances lipid accumulation during the macrophage foam cell formation. (A)** Human THP-1 macrophages. **(B-D)** THP-1 macrophages were pre-treated with oxLDL (50 μg/ml) for 24 h and exposed to LPS (1 μg/ml) in RPMI1640 with 10% FBS for different periods (8, 16, and 24 h). Lipid droplets were visualized by Oil Red O staining. **(E, F)** The levels of triglcyceride (TG) and total cholesterol (TC) were evaluated. Data were presented as means ± S.E. of μM of triglyceride or cholesterol per milligram protein from 3 independent experiments. *significant difference from LPS treatment P < 0.05; **, P < 0.01.

To examine the effect of LPS on macrophage lipid accumulation during foam cell formation, human THP-1 macrophage-derived foam cells were incubated with LPS (1 μg/ml) aggregates for a different periods (0, 8, 16, and 24 h), followed by visualization of lipid laden in macrophages with Oil Red O staining. As shown in Figure 1A-D, the number of lipid droplets in the cytoplasm of cells treated with LPS for 24 h were obviously induced compared to the vehicle treated cells. Then, we harvested the cells to determine cellular total cholesterol and triglyceride content. Consistent with Oil Red O staining, measurement of intracellular lipid contents indicated that the level of triglyceride and total cholesterol (Figure 1E and F) were markedly increased after LPS incubation. These data showed that LPS augmented lipid accumulation during the foam cell formation.

### Autophagy was activated during LPS-induced foam cell formation

**Figure 2:**
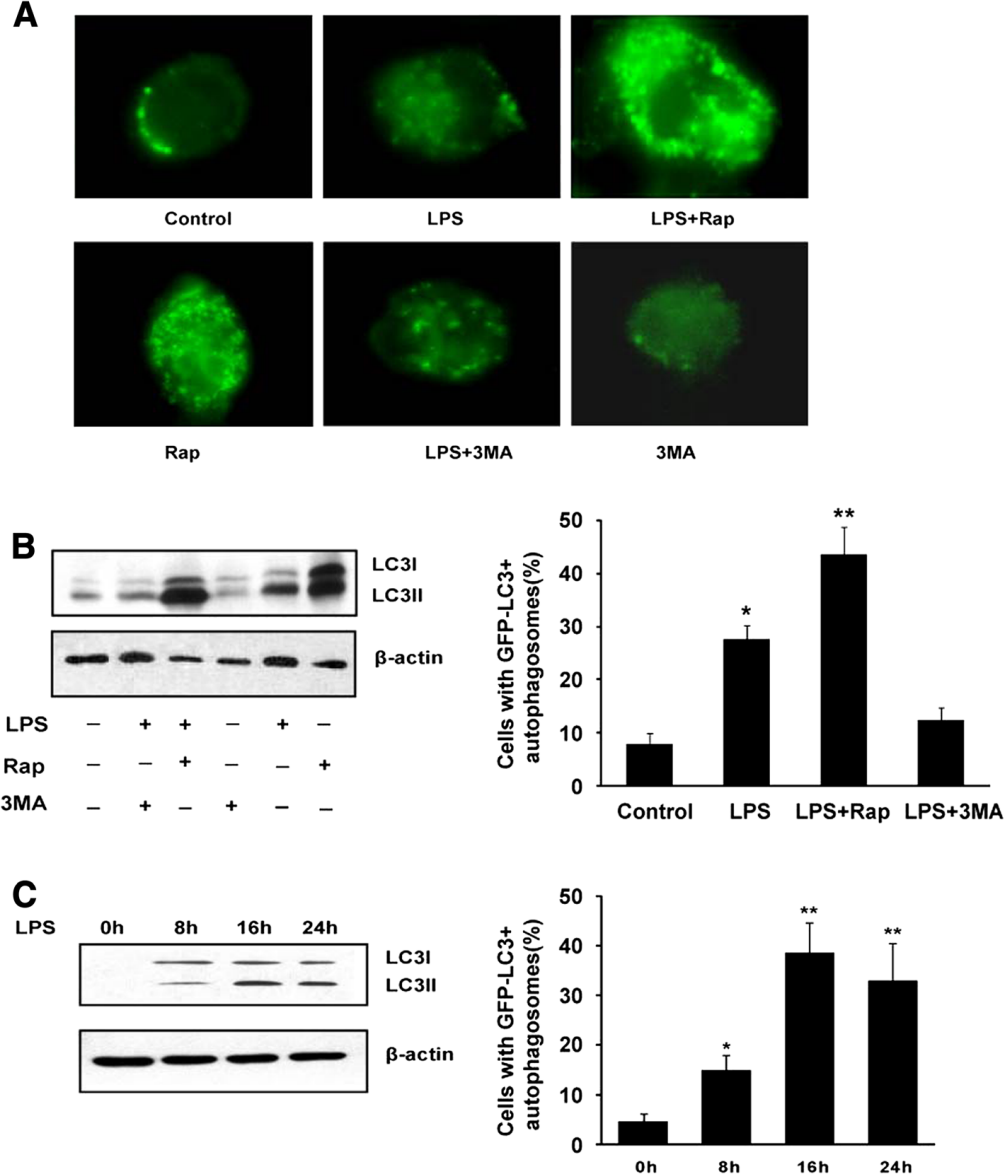
**LPS increases the autophagic activity during the human macrophage foam cell formation.** THP-1 macrophages were incubated in the absence (control) or presence of LPS (1 μg/ml), LPS plus rapamycin (Rap, an autophagy activator, 10 μM), Rap alone, LPS plus 3-methyladenine (3MA, an autophagy inhibitor, 10 μM) or 3MA alone as indicated (n = 3). **(A)** THP-1 human macrophages stably expressing GFP-LC3 were treated with LPS (1 μg/ml) for the indicated time points. **(B)** The levels of LC3II and LC3I were detected with specific antibodies by using immunoblotting and the percentage of cells with autophagosomes was calculated. **(C)** Representative immunofluorescence images with GFP-LC3^+^ in THP-1 human macrophages after exposure to 1 μg/ml LPS for the indicated time points (0, 8, 16, and 24 h) and the percentage of cells with autophagosomes were calculated. Data represent mean ± S.E. from 3 independent experiments. *significant difference from LPS treatment *P* < 0.05; **, *P* < 0.01.

To evaluate whether autophagy was significantly different after LPS treated, we analyzed distribution of LC3, typical of autophagosomes [13,14]. Autophagy formation can be visualized with an electron microscope, which is by far the most confirmative analysis for autophagy. LC3 is recruited to autophagosome membranes during autophagy, and GFP-LC3 has been used as a marker for autophagy induction [15]. Interestingly, by transfection with GFP-LC3, we found that LPS exposure resulted in the redistribution of GFP-LC3 from a diffuse to a punctate pattern, and induced a significant increase in the percentage of cells with GFP-LC3 autophagosomes (Figure 2A). Autophagic activity in human THP-1 macrophages was either stimulated with Rap, an inhibitor of mTOR, and inhibited by using 3MA, an inhibitor of autophagy. Exposure of THP-1 macrophages to LPS increased Rap induced the LC3-GFP-labeled autophagosomes whereas 3MA decreased the number of LPS-induced autophagosomes in macrophages (Figure 2A). When autophagy occurs, cytosolic LC3 (LC3-I) is modified to its membrane-bound form (LC3-II), which is located on preautophagosomes and autophagosomes and thus commonly used as an autophagosomal marker [15]. An increased LC3II/LC3I ratio indicates enhanced autophagy activity. Consistently, Rap increased the ratio of LC3II/LC3I whereas 3MA decreased the LC3II/LC3I ratio. Exposure to LPS increased the ratio of LC3II/LC3I and the GFP-labeled autophagosomes (Figure 2B). Moreover, the time-course (0, 8, 16, and 24 h) experiments revealed that the percentage of cells expressing autophagosomes was maximal at 16 h after LPS stimulation (Figure 2C). These data indicate that LPS might activate autophagy during foam cell formation.

### Modulation of autophagic activity can alter the accumulation of lipid droplets during the foam cell formation

**Figure 3:**
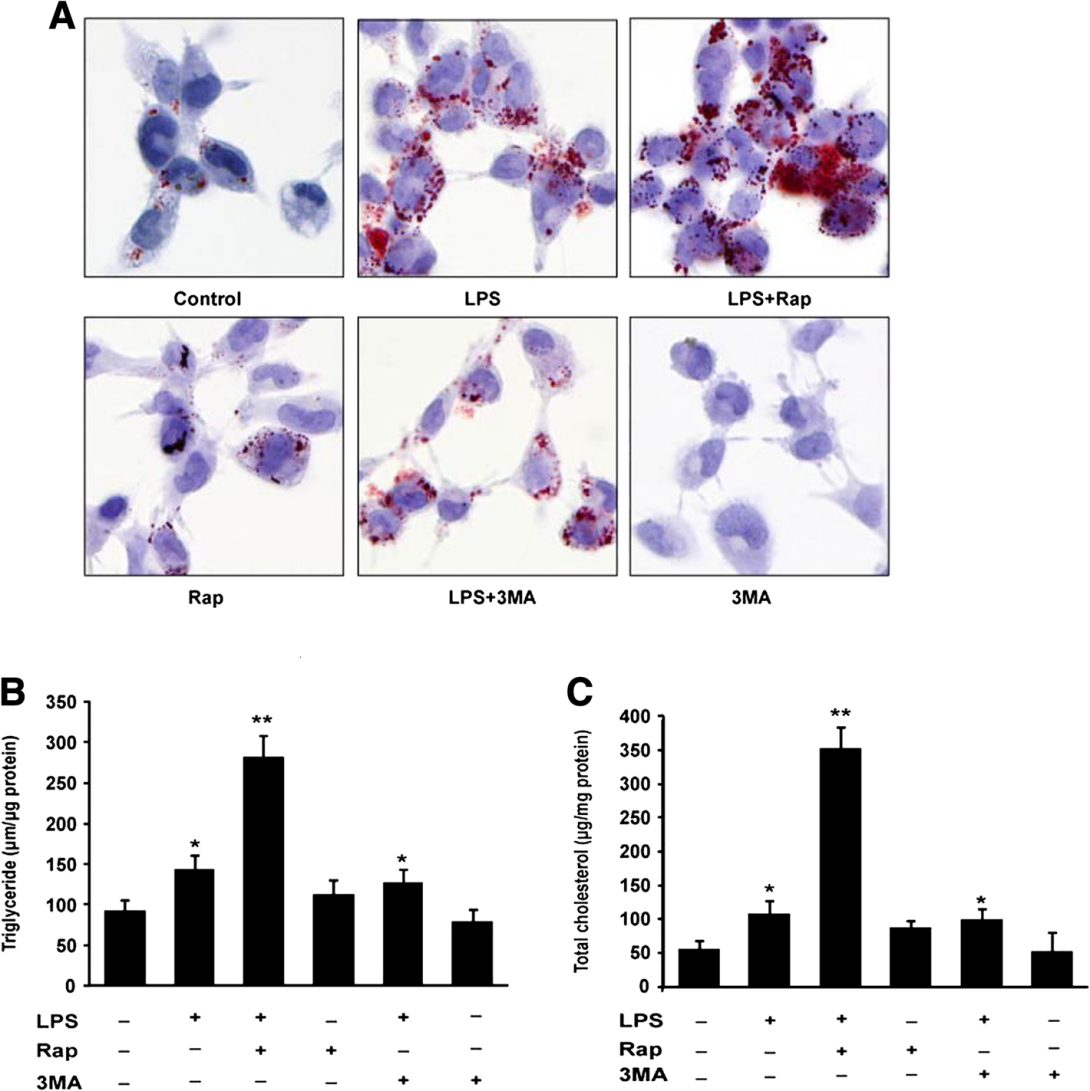
**Modulation of autophagic activity alters the accumulation of lipid droplets induced by the exposure to LPS in THP-1 macrophages. (A)** THP-1 macrophages were pre-treated with oxLDL (50 μg/ml) for 24 h, then incubated in the absence (control) or presence of 1 μg/ml LPS, LPS plus Rap (10 μM), Rap alone, LPS plus 3-methyladenine (3MA, an autophagy inhibitor, 10 μM) or 3MA alone for 24 h as indicated (n = 3). Cells were fixed and stained with Oil Red O to visualize the lipid droplets. **(B,C)** The levels of total cholesterol and triglcyceride were evaluated by enzymatic colorimetric assays. Results were presented as mean ± SEM of 3 independent experiments, each in triplicate. *significant difference from LPS treatment *P* < 0.05; **, *P* < 0.01.

Autophagy has recently been implicated in the control of lipid accumulation [16-20]. To examine the direct effect of autophagy during lipid accumulation in macrophages, autophagy activity in human THP-1 macrophages was either stimulated with Rap or inhibited by using 3MA. As shown in Figure 3A, lipid accumulation was increased by the exposure to LPS, and the increase was enhanced by Rap. In contrast, inhibition of autophagy with 3MA prevented both the basal and the LPS-induced lipid accumulation (Figure 3A), whereas Rap alone did not alter the level of total cholesterol or triglyceride level. In contrast, inhibition of autophagy with 3MA decreased triglyceride in macrophages that were either treated with LPS or not (Figure 3B). Together, these results demonstrate that autophagic activity on lipid droplets plays an important role in the accumulation of lipids during foam cell formation.

### The level of ADRP is correlated with the autophagic activity in LPS-treated macrophages

**Figure 4:**
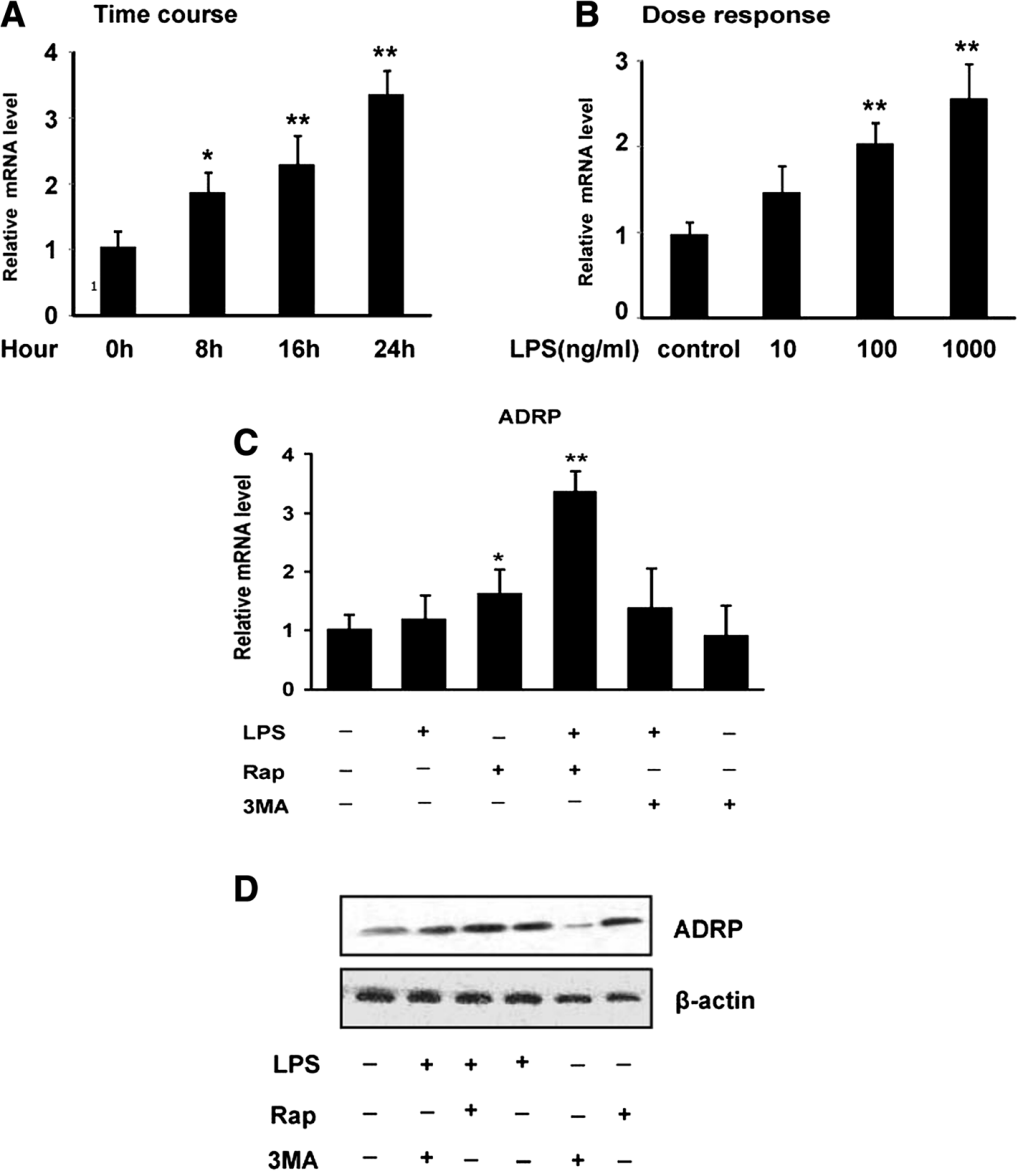
**Autophagy altered the expression of ADRP in LPS-stimulated THP-1 macorphages. (A, B)** Macrophages were incubated with LPS for a different time (0, 8, 16, and 24 h) or with different amounts of LPS (10-1000 ng/ml) for 24 h. Real-time PCR analysis *ADRP* gene expression. **(C)** THP-1 cells were treated with 1 μg/ml LPS in the absence or presence of Rap (10 μM), or 3MA (10 μM) as noted for 24 h. The level of *ADRP* was then quantified by real-time PCR. **(D)** The levels of ADRP and β-actin proteins were detected by using immunoblotting with specific antibodies. Results were presented as mean ± S.E. of 3 independent experiments. *significant difference from LPS treatment *P* < 0.05; **, *P* < 0.01.

ADRP is a lipid droplet-associated proteins that plays a critical role in the homeostasis of cytosolic lipid droplets in various types of cells [7]. ADRP is also coated on lipid droplets (LDs) in the macrophage and foam cells, and its levels is directly correlated with cellular neutral lipid content in the foam cells of atherosclerosis [21,22]. To examine whether ADRP is involved in LPS-induced autophagy, macrophages were incubated with LPS aggregates for a different time (0, 8, 16, and 24 h) or for 24 h with different amounts of LPS (10–1000 ng/ml), the total RNA was then extracted, and gene expression was measured by real-time PCR analysis. As shown in Figure 4A and B, exposure to LPS stimulated ADRP in a time-and dose-dependent manner. Furthermore, treatment with Rap upregulated the levels of ADRP. In contrast, 3MA inhibited ADRP protein levels as shown by western blotting analysis (Figure 4C and D). These results demonstrate that the level of ADRP in macrophages is positively correlated with the autophagic activity after treatment with LPS.

### ADRP enhances autophagy in the LPS-induced macrophage foam cell

**Figure 5:**
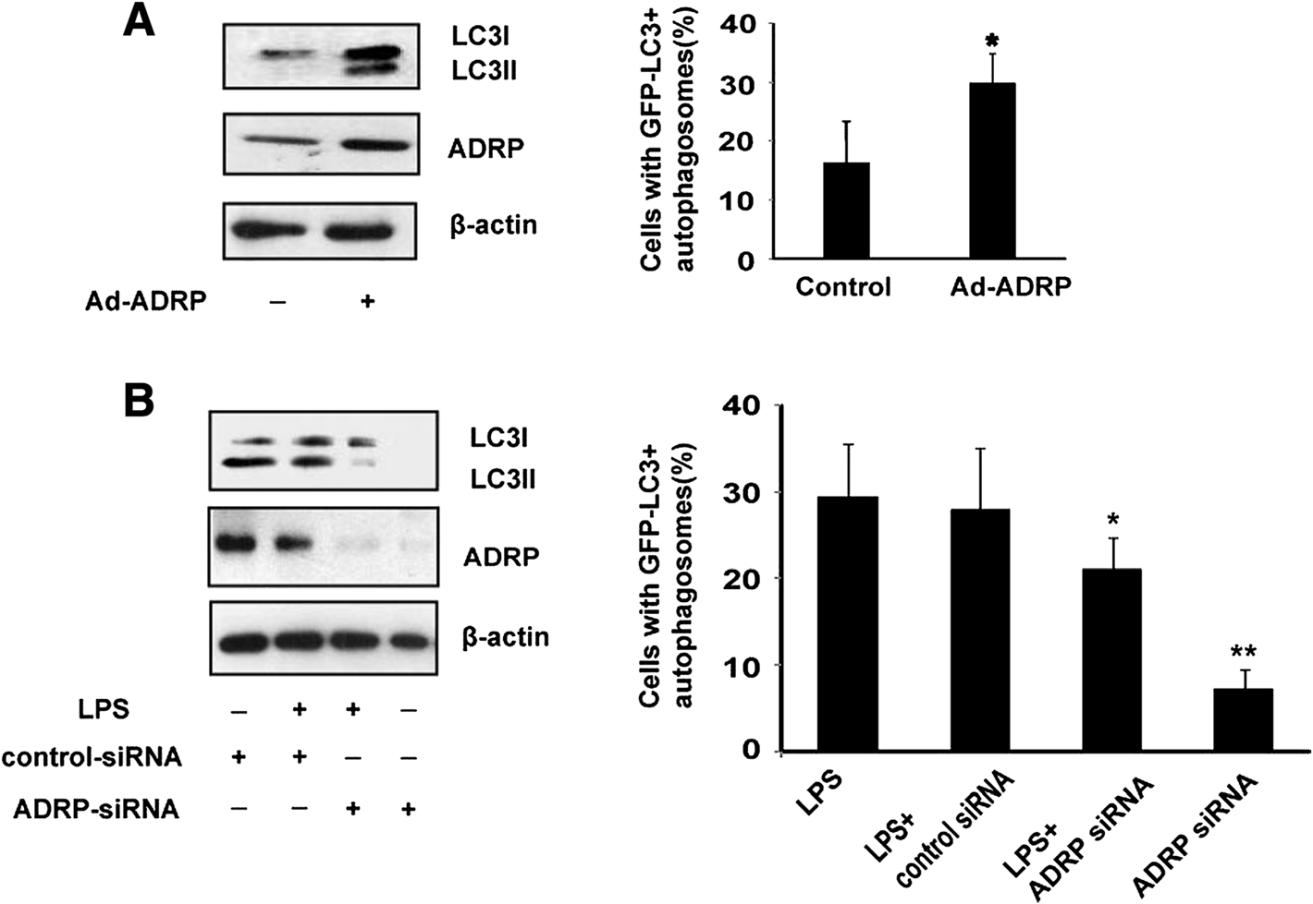
**ADRP overexpression increases the LPS-induced autophagic activity, while ADRP knockdown downregulates the autophagic activity in THP-1 macrophages. (A)** ADRP overexpression by adenoviral gene transfer augmented LPS-induced LC3 protein levels as determined by western blotting and the percentage of cells with autophagosomes were calculated. **(B)** Specific knockdown of ADRP with ADRP siRNA prior to LPS stimulation of THP-1 cells inhibited the LC3-II/I ratio as determined by western blotting and the percentage of cells with autophagosomes was calculated. Results were presented as mean ± SEM of 3 independent experiments, each in triplicate. *significant difference from LPS treatment *P* < 0.05; **, *P* < 0.01.

To determine the role of ADRP in the LPS-induced autophagy during the foam cell formation, ADRP was overexpressed in THP-1 macrophages by transfection with pCMV5-ADRP plasmid. We confirmed that the LC3-II/LC3-I ratio increased when ADRP was overexpressed in macrophages, indicating that overexpressed ADRP could enhance LPS-induced autophagy (Figure 5A). Furthermore, small interfering RNA (siRNA) against ADRP was used to knockdown ADRP in THP-1 cells stably expressing GFP-LC3. Subjected to LPS treatment and evaluated for autophagy, we found that knockdown of ADRP with specific siRNA inhibited LPS-induced LC3-II levels in comparison with a scrambled nontargeted siRNA (Figure 5B). In summary, ADRP could enhance the LPS-induced autophagic activity on lipid droplets in THP-1 macrophages.

## Discussion

● The data in this study focus on the functional relationships between autophagy and lipid metabolism. We found that autophagy at least partially augmented LPS-induced foam cell formation owing to its effect on the induction of ADRP.

Foam cell formation is a hallmark of the initial stage of atherosclerosis. The activation of macrophages by LPS has been shown to increase fat accumulation (Figure 1). In fact, studies have demonstrated that LPS influences many aspects of macrophage-derived foam cell formation, such as the promotion of LDL oxidation [23], lipid uptake [24], up-regulation of macrophage fatty acid transport and inhibition of cholesterol efflux [25,26].

Recently, a study has demonstrated that blocking autophagy in cultured preadipocytes decreases the cellular triglyceride content and levels of key adipogenic transcription factors CEBP-α (CCAAT/enhancer-binding protein alpha), CEBP-β (CCAAT/enhancer binding protein beta ) and PPAR-γ [27]. However, little is known about the role of autophagy in atherosclerosis. In this study, we demonstrate that autophagy is activated in LPS-induced macrophage foam cells (Figure 2). Here, we just focus on the autophagy involved in the uptake of lipid and lipid droplet formation in macrophage-derived foam cells. In this work, the activation of autophagy enhanced levels of intracellular cholesterol and triglyceride while inhibition of autophagy could block the accumulation of lipid droplets in macrophages (Figure 3). Consist with these results, an increase rather than a decrease in foam cell formation would be expected in macrophages undergoing autophagy because protein degradation, as well as the decline of protein synthesis, in autophagic cells readily blocks the utilization of lipids for lipid–protein conjugation, which in turn results in the formation of lipid droplets [28].

ADRP is the major macrophage LD coat protein, and its levels are directly related with cellular neural lipid content [29]. Whole-body as well as macrophage specific ADRP deficiency inhibits foam cell formation and protects against atherosclerosis development [29]. Despite ADRP is an absolute LD coat protein, and was also detected in lysosomes in macrophage foam cell, suggesting that ADRP may play an important role in autophagy [30]. Our results also support a possible role for ADRP in LPS-induced autophagic signaling. Overexpression of ADRP leads to augment of autophagy, which has been shown to increase autophagic signaling. Moreover, inhibition of ADRP can suppress the activation of autophagy (Figures 4, 5). Interestingly, ADRP overexpression prevented cholesterol efflux to apolipoprotein A-I (ApoA-I) [31]. One possible explanation is that ADRP may be localized around cytosolic lipid droplets in the macrophages, protecting them from the activity of cholesterol esterases and thereby reducing the availability of free cholesterol (FC) for efflux. Thus, autophagy is activated in response to a lipid challenge. These results indicated that ADRP could protect LDs against the lipolysis mediated by autophagy and lysosomes in the LPS-induced macrophage foam cell.

In conclusion, these data link autophagy and lipid metabolism, two separate pathways that are both activated by LPS. Although further studies are required to establish a correlation between the regulation of autophagy in macrophages and atherosclerosis, our results suggest that the autophagic activity may be involved in the accumulation of lipid droplets in macrophage foam cells. Furthermore, our data indicate that ADRP facilitates autophagic activity in the LPS-induced macrophage foam cells. Summarily, these results are helpful for us to understand the role of autophagy in the development of atherosclerosis.

## Competing interests

There are no competing interest in this study.

## Authors’ contributions

XYF and YY carried out the study design, data collection and analysis, and drafted the manuscript. CW and JF carried out the autophagy assays. ZYY and XMZ participated in the real-time PCR experiment. WS and PZH constructed ADRP-siRNA and pCMV5-ADRP plasmids. PFZ participated in the measurement of intracellular cholesterol and triglyceride levels in foam cells. JY conceived of the study, and participated in its design and coordination and helped to draft the manuscript. All authors read and approved the final manuscript.
